# Case report: A novel 11-bp deletion in exon 11 causing a frameshift in the C-terminal of the *ALAS2* gene leading to X-linked sideroblastic anemia—a family study

**DOI:** 10.3389/fmed.2024.1452873

**Published:** 2025-02-10

**Authors:** Salam Al kindi, Altaf Al-Mamari, Shoaib Al-Zadjali, Mohamed Al-Rawahi, Ali Al Madhani, Anil V. Pathare

**Affiliations:** ^1^Department of Haematology, Sultan Qaboos University Hospital, Muscat, Oman; ^2^College of Medicine & Health Sciences, Muscat, Oman; ^3^Department of Medicine, Sohar Hospital, Sohar, Oman

**Keywords:** sideroblastic anemia, XLSA, X-linked, *ALAS2*, congenital

## Abstract

X-linked sideroblastic anemia (XLSA) (MIM 300752) is the most common genetic form of sideroblastic anemia, a heterogeneous group of disorders characterized by iron deposits in the mitochondria of erythroid precursors. It is due to mutations of the erythroid-specific enzyme *ALAS2*, the first enzyme of the heme biosynthetic pathway. Herein, we report a novel 11-bp deletion in exon 11 leading to a frameshift in the C-terminal region of the *ALAS2* gene with a non-functional longer polypeptide of 614 amino acids leading to a loss-of-function mutation manifested as an X-linked sideroblastic anemia phenotype. The proband was a 29-year-old man with moderately severe microcytic hypochromic anemia with splenomegaly and increased ring sideroblasts in the bone marrow with considerable iron overload. Sanger sequencing documented a missense mutation leading to a frameshift with an elongated polypeptide of 614 AA instead of the normal 587 AA protein c.1743_1753 del (p.Gln581Hisfs*35). This mutation affected the interaction with cofactor pyridoxal 5′-phosphate since the patient’s hemoglobin improved with oral administration of pyridoxine tablets. His iron overload also responded to sustained oral iron chelation therapy with deferasirox. The screening of the entire family’s kindred revealed that two other male siblings were also hemizygous for the same mutation with hypochromic microcytic anemia and tissue iron overload, whereas, three female siblings and their mother were heterozygous for the mutant allele. They did not have anemia or iron overload.

## Introduction

Congenital sideroblastic anemias (CSAs) are a heterogeneous group of disorders that arise as a result of iron deposition in the mitochondria inside the bone marrow erythroid precursors appearing as ringed sideroblasts (1, 2). This leads to ineffective erythropoiesis, increased iron overload, and hypochromic microcytic anemia. The most common cause of CSA is X-linked sideroblastic anemia (XLSA) (OMIM: 300752), which is caused by mutations of the erythroid-specific delta-aminolevulinate synthase gene (5′-aminolevulinate synthase 2; *ALAS2*) located at Xp 11.21. *ALAS2* encodes the first enzyme in the heme biosynthetic pathway, resulting in deficient heme synthesis (3, 4).

Human *ALAS2* protein consists of 587 amino acids, and its carboxyl(C)-terminal region consists of 33 amino acids (5). ALAS is a family of pyridoxal 5′-phosphate (PLP; a metabolite of vitamin B6) dependent enzymes, and crystallographic evidence suggests that ALAS is a tightly interlocked homodimer (6). Each monomer consists of three domains, all of which participate in dimerization: an N-terminal domain, a central (catalytic domain that contributes most of the dimer interface), and a C-terminal domain. The C-terminal domain includes a three-stranded, antiparallel *β*-sheet contacting both N-terminal and catalytic domains of the same monomer and three *α*-helices contacting the N-terminal domain.

At least 99 mutations that cause X-linked sideroblastic anemia have been identified in the Human Gene Mutation Database (HGMD) (7). Almost all of these mutations change a single amino acid in the erythroid *ALAS2*. These changes impair the activity of the enzyme, which disrupts the normal production of heme in developing red blood cell precursors leading to the loss-of-function effect. The majority of mutations at the C-terminal region have also been associated with a loss-of-function and X-linked sideroblastic anemia phenotype. However, although XLP was originally reported as X-linked dominant with 100% penetrance in males and females, subsequent studies confirm that the previous dominant classification of XLP is inappropriate and genetically misleading, as the disorder is more appropriately designated as XLP or XLEPP (OMIM 300752) (8).

In this study, we describe a novel mutation in the C-terminal of the *ALAS2* gene in a man, in his late twenties, who was incidentally detected and diagnosed to have an X-linked sideroblastic anemia phenotype.

## Case report

A 29-year-old Omani male (proband), a teacher by profession, was referred to our health facility for evaluation of anemia with high ferritin and splenomegaly. All findings were incidental as he went for a job interview, and the prerequisite baseline blood investigations were performed documenting anemia. He had no complaints and was doing well. He has a family history of anemia in his siblings but without a definite diagnosis. There was no past history of surgery or previous hospital admissions. He had never received any blood transfusions. His parents were not consanguineous. Clinical examination revealed a young, well-built, Omani male, with mild pallor, no jaundice, and no lymphadenopathy, but with an enlarged, palpable spleen 3 cm below the left costal margin.

Laboratory investigation revealed the presence of microcytic hypochromic anemia with hemoglobin (Hb) 8.84 g/dL, mean corpuscular volume (MCV) of 48 fL, mean corpuscular hemoglobin (MCH) of 14.5 pg., and red cell distribution width (RDW) of 34.3%. The white blood cell (WBC) count was 4.79 × 10^9^/L, and the platelet count was 360 × 10^9^/L. Blood film examination showed significant anisopoikilocytosis with target cells, teardrops, and pencil cells with elliptocytes. Leukocyte and platelet morphology was normal. Bone marrow examination revealed marked erythroid hyperplasia with dysplastic features including irregular cytoplasmic outline, chromatin clumping, and bi-nucleated forms. Iron stain showed increased iron granules in erythroid precursors with many ring sideroblasts. Granulocytes and megakaryocytes appeared normal. Cytogenetics workup showed a normal karyotype. Hemoglobin electrophoresis revealed Hb A at 96.6%, Hb A2 at 2.4%, and Hb F at 0.2%. Serum iron was 45 umol/L, serum transferrin was 1.96 g/L, and transferrin saturation was 91.4%. Serum ferritin was 5,836 ng/mL, and the liver function test revealed mildly elevated transaminases with alanine aminotransferase (ALT) at 120 u/L and aspartate aminotransferase (AST) of 65 u/L. Serology for Hepatitis B and C was negative. After the initial presentation of the proband, blood samples of all the patient’s family members were obtained following informed consent, and the results are given in Table 1.

**Table 1:** Demography, full blood count, serum ferritin, and *ALAS2* genotype status in the kindred.

Sr. No	Age, yrs	Gender	Hb g/dL	MCV fl	MCH pg	RDW %	S. Ferritin ng/ml	*ALAS2* genotype/mutation
I-II	61	Female	12.8	84.6	26.4	15.4	17.2	Heterozygous
II-1	33	Female	12.2	78.1	24.7	16.6	18.9	Heterozygous
II-2,P	30	Male	7.4	47.4	13.2	34.5	5,836	Hemizygous
II-3	29	Male	8.8	48.1	14.5	34.2	2,716	Hemizygous
II-4	27	Male	15.5	79.8	25.7	12.6	22.4	*ALAS2* wild type
II-5	22	Female	12.7	75.3	23.1	15.7	34.1	*ALAS2* wild type
II-6	21	Female	12.3	75.1	12.8	14.2	15.7	*ALAS2* wild type
II-7	21	Female	10.3	62.8	16.5	17.2	14.2	*ALAS2* wild type
II-8	19	Male	6.8	49.4	13.5	35.7	1,165	Hemizygous
II-9	17	Female	12.1	80.1	21.1	16.1	26.6	Heterozygous
II-10	15	Female	12.9	76.3	19.6	15.3	16.2	Heterozygous
II-11	12	Female	13.7	80.5	23.9	13.8	54.2	Heterozygous

Reference ranges. Hb in males (11.5–15.5 g/dL), Hb in females (11.0–14.5 g/dL), MCV (78–95 fl), MCH (26–33 pg), RDW (11.5–16.5%), S ferritin (15–50 ng/mL), P, proband.

**Figure 1:**
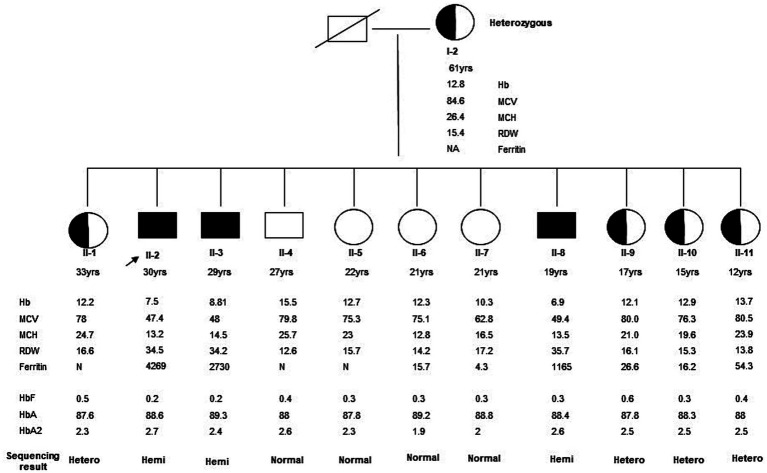
Family Pedigree of the kindred was evaluated in this study. The arrow indicates the proband. The affected male (II-2) and his two male siblings (II-3 and II-4) showed a hemizygous *ALAS2* mutation. This is a novel 11-bp deletion in exon 11 causing a frameshift in the C-terminal of the *ALAS2* gene leading to X-linked sideroblastic anemia. Mother (I-2) and four female siblings (II-1, II-9, II-10, and II-11) are heterozygous carriers, whereas the remaining 4 siblings (three females II-5, II-6, and II-7, and a male II-8) are normal with wild type *ALAS2* gene.

**Figure 2:**
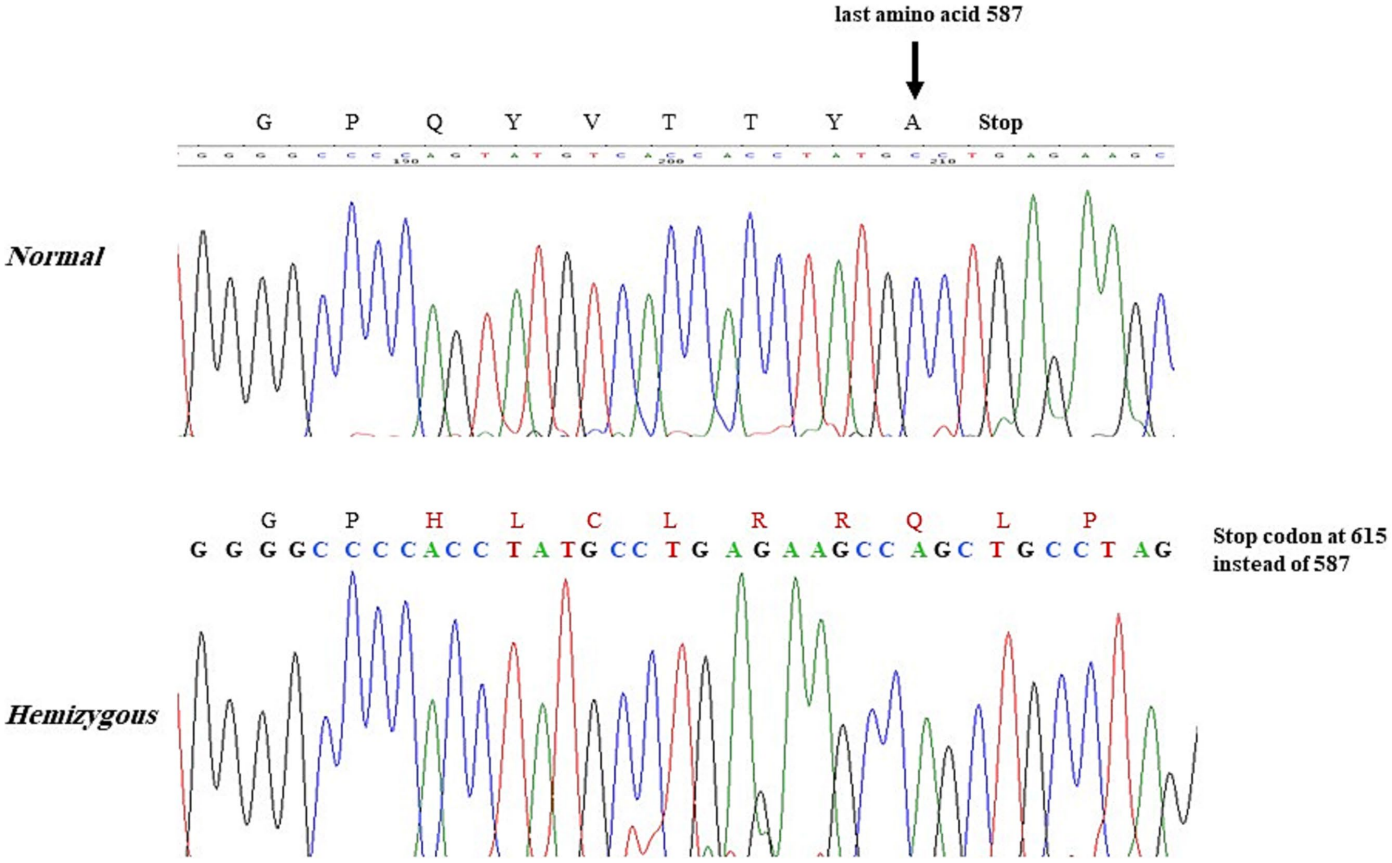
Proband and 2 male siblings showing the 11-bp deletion in the *ALAS2* gene leading to the amino acid change Gln(Q) to His(H) at position 581 with frameshift causing a stop codon at 615 instead of 588.

DNA/Molecular analysis of the *ALAS2* gene was performed by obtaining high molecular weight DNA from the whole blood using the QIAamp DNA Blood Mini Kit (Qiagen Inc., Valencia, CA, and USA). The 11 exons of the *ALAS2* gene and the 250 base pair (bp) promoter region of the *ALAS2* gene were amplified using polymerase chain reaction (PCR) with primers as reported by Furuyama K et al. (9). Each 20 μL PCR contained 200 nM of each primer, 100 ng of template DNA, and Fast PCR master mix (Applied Biosystems, Life Technologies, and Foster City, CA, USA). The PCRs were carried out in fast mode (20 min) using fast PCR 9800 (Applied Biosystems, Life Technologies). The hemizygous PCR products were then purified using ExoSAP (USB, Cleveland, OH, USA) and then cycle-sequenced on the ABI 3100 analyzer using the BigDye Terminator Cycle Sequencing Kit v3.1 (Applied Biosystems, Life Technologies). Sanger sequencing of the *ALAS2* gene was performed on the patient and his family members, leading to the identification of a missense mutation. A G to C transition in exon 11 of the *ALAS2* gene, caused a Glycine (Gly) to Histidine (His) substitution at the amino acid position 581 [NM_000032.5 (*ALAS2*): c.1743_1753del, (p. Gln 581 His fs*35)], causing a frameshift at the C-terminal of the *ALAS2* gene in the proband (Figure 1). The proband and two other male siblings (II-3 and II-8) were found to have a hemizygous 11-bp deletion in exon 11, resulting in a longer polypeptide of 614 amino acids (Figure 2). Proband’s mother (I-2) and his four sisters (II-1, II-9, II-10, II-11) were found to be heterozygous (for the same mutation with no anemia or iron overload). Furthermore, his three other sisters and a male sibling showed normal germline *ALAS2* wild type with normal full blood count and serum ferritin levels.

The patient was treated with pyridoxine 100 mg tablets daily, with significant improvement in the hemoglobin levels (from 7.3 to 11.3 g/dL). In addition, hypochromic microcytosis showed some improvement (MCV-60; MCH-18.1). He has been on iron chelation treatment using deferasirox 1,000 mg/day for the past 5 years. His serum ferritin decreased from 4,269 to 506 ng/mL over the 5-year follow-up period. His transferrin saturation has now become within the normal range, from the initial high value of 91%. His MRI for cardiac and liver iron load revealed a normal cardiac MRI T2* range of 28.9–39.8, but significant liver iron overload was observed, with an initial value of 21.4 g/dw, which reduced to 3.5 g/dw following iron chelation treatment. Furthermore, the elevated liver transaminases, AST and ALT, returned to normal levels. The patient also showed evidence of osteopenia with low bone mineral density, with a T-score of-2.3 and *Z*-score of −1.9 on NM bone densitometry (DEXA scan), was performed and showed evidence of osteopenia with low bone mineral density, showing T-score of-2.3 and Z-score of −1.9. This has returned to normal levels for age following treatment with bisphosphonates (Zoledronic acid 5 mg infusions yearly).

## Discussion

Our proband showed considerable improvement in his hemoglobin levels following the administration of oral pyridoxine supplementation. Hemoglobin response to initiation of pyridoxine therapy is usually time dependent. It is also influenced by the intrinsic severity of the defect and possible secondary causes, such as iron toxicity, leading to a variable response, as reported in the literature (10). Nevertheless, among the mutations associated with hematological response to pyridoxine, there is no obvious relationship between the mutation and the clinical response (11). It is believed that PLP is reversibly bound to Lysine 391 and this binding is absolutely essential for enzymatic activity and also crucial for its stability, maintaining a conformation that is optimal for substrate binding and the catalytic activity of the enzyme (12). Additional factors likely to affect the clinical outcome is the degree to which the enzyme with low activity can be compensated by an increased amount within the mitochondria to which the low amount of enzyme can be compensated for by increased specific activity (11). Thus, pyridoxal phosphate (oral pyridoxine) is apparently necessary to stabilize a functionally adequate enzyme. This explains the high specific activity mediated by the addition of pyridoxine to support better hemoglobinization of the patient’s RBCs in XLSA. In fact, the responsiveness to pyridoxine seen in XLSA also helps to distinguish it from other congenital causes of inherited sideroblastic anemia with mutations in *SLC25A38*, *ABCB7*, *GLRX5*, *PUS1*, and *SLC19A* genes (3). Furthermore, pyridoxine-responsive XLSA is known to have a variable age of onset at presentation and our proband is no exception. He was anemic but did not present with specific symptoms and was only diagnosed incidentally. Clearly, these patients are not anemic at birth, and although they have a congenital genetic defect, early presentation is unlikely unless the defect itself is a severe one (11). Decreased/fluctuating levels of plasma pyridoxine are generally observed in the elderly and manifest with clinical variability of pyridoxine-responsive XLSA (13).

The mutation in our proband manifested with a phenotypic expression of X-linked sideroblastic anemia. The proband and his two siblings were noted to be hemizygous for the presence of a novel 11-bp deletion in exon 11 causing a frameshift at the C-terminal of the *ALAS2* gene. This produced a large polypeptide (614 AA instead of 587 AA) with reduced heme production. Thus, it needs to be emphasized that even a longer protein can give rise to XLSA just as stop mutations give rise to XLEPP (7). The heme synthesis deficiency leads to ineffective erythroid maturation, resulting in erythroid hyperplasia in the bone marrow and subsequent iron deposition in the mitochondria, which is observed as ringed sideroblasts on Prussian Blue staining (11). Thus, despite a high iron delivery as seen with the abnormally high transferrin saturation (91%) in the proband, there is a persistently increased iron absorption by the usual feedback mechanisms as the patient showed significant iron overload in the absence of any external blood transfusions or iron ingestion. It is, thus, a direct effect of non-utilization of iron in the heme production pathway owing to the lack of activity/function of the *ALAS2* enzyme due to the defective genetic mutation that was demonstrated in these kindred. Thus, although the anemia of pyridoxine-responsive XLSA can be treated with pyridoxine, iron overload remains the main complication, and without appropriate treatment, diabetes, liver, and heart failure or osteoporosis can occur, as the latter two complications were noted in our proband (11). Iron overload essentially occurs owing to increased absorption, but other genetic causes such as mutations in the *HFE* gene may also complicate the severity of iron overload, although this was ruled out in our proband. Furthermore, our patient has responded remarkably well to the oral iron chelator deferasirox 1gm daily.

The carboxyl(C)-terminal region of the *ALAS2* gene contributes to 33 amino acids and plays an essential role in the interaction between the N-terminal domain and catalytic domain to facilitate the enzymatic activity of *ALAS2*. Many well-known mutations in the C-terminal region exhibit different phenotype expressions, with the majority being missense or nonsense point mutations at the C-terminal region, which result in a loss-of-function (Table 2). However, Ducamp et al. (7), reported a frameshift deletion at the C-terminal resulting in a gain-of-function with phenotypic expression EPP. It is believed that the inhibitory effect of the C-terminal region on the enzymatic activity of this mutation results in a gain-of-function leading to an increase in the enzyme activity, with increased levels of protoporphyrin causing cutaneous photosensitivity.

**Table 2:** Missense/nonsense mutations and deletions in the C-terminal region of the *ALAS2* gene [NCBI Reference Sequence: NM_000032.5].

Mutation	Codon change	Variant ID	Amino Acid change	HGVS consequence	Outcome	Reference
R559H	CGC-CAC	X-55009268-G-C	Arg-His	p.Arg559His	XLSA	Pereira (14) Hum Genet 115, 533
R560H	CGT-CAT	X-55009265-G-A	Arg-His	p.Arg560His	XLSA	Cazzola (15) Blood 100, 4,236
V562A	GTA-GCA	X-55009260-T-C	Val-Ala	p.Val562Ala	XLSA	Harigae (16) Int J Hematol 92, 425
H563L	CAC-CTC	X-55009285-A-T	His-Leu	p.His263Leu	XLSA	Liu (17) Haematologica 98, e158
E565L	GAG-AAG	X-55009251-G-A	Glu-Lys	p.Glu565Leu	XLSA	Liu (17) Haematologica 98, e158
M567I	ATG-ATA	X-55009246-G-A	Met-Ile	p.Met567Iso	XLSA	Harigae (16) Int J Hematol 92, 425
M567V	ATG-GTG	X-55009244-A-G	Met-Val	p.Met567Val	XLSA	Bishop (18) J Biol Chem 287, 28,943
S568G	AGT-GGT	X-55009241-A-G	Ser-Gly	p.Ser568Gly	XLSA	Harigae (19) Br J Haematol 106, 175
R572H	CGT-CAT	X-55009229-G-A	Arg-His	p.Arg572His	XLSA	Ducamp (20) Hum Mutat 32, 590
Q581H	CAG-CAC	c.1743_1753del, p. (Gln581Hisfs*35)	11-bp del	p.Gln581His*35	XLSA	Present Study Alkindi et al. #
T586P	TAT-TTT	X-55009187-A-T	Tyr-Phe	p.Tyr586Phe	XLDPP	To-Figueras (21) Blood 118, 1,443
Q548X	CAG-TAG	X-55009300-C-TAG	Gln-STOP	p.Gln548 Stop	XLDPP	Bishop (22) Mol Med 19, 18

Key: These missense or nonsense mutation details were obtained from the Human Gene Mutation Database (HGMD) (7) as well as the gnomAD database (https://gnomad.broadinstitute.org/).

In conclusion, we report a novel 11-bp deletion in exon 11 causing a frameshift in the C-terminal of the *ALAS2* gene leading to X-linked sideroblastic anemia XLSA phenotype. The phenotype of XLSA in this proband was mild to moderate, characterized by hypochromic microcytic anemia, iron overload, and ring sideroblasts in the bone marrow. The diagnosis was confirmed by molecular testing of the *ALAS2* gene, and the response to oral pyridoxine further confirmed the diagnosis of pyridoxine-responsive XLSA. This patient demonstrated a good response to pyridoxine supplementation and iron chelation therapy.

## Data Availability

The original contributions presented in the study are included in the article/supplementary material, further inquiries can be directed to the corresponding author/s.
